# Saponins enhance the stability and cost-efficiency of human embryonic stem cell culture

**DOI:** 10.1186/s13619-024-00220-y

**Published:** 2025-01-21

**Authors:** Jingyi Shi, Mei Wu, Shi Fang, Zhuo Liu, Huihui Liu, Ying Zhao, Linlin Liu, Zhicheng Shao

**Affiliations:** 1 Department of Neurology, Zhongshan Hospital, Institute for Translational Brain Research, State Key Laboratory of Medical Neurobiology, MOE Frontiers Center for Brain Science, Fudan University, Shanghai, 200032 China; 2 School of Pharmacy, Xinxiang Medical University, Xinxiang, 453003 China

**Keywords:** Saponins, Human embryonic stem cells, Culture medium, Brain organoid

## Abstract

The cultivation and differentiation of human embryonic stem cells (hESCs) into organoids are crucial for advancing of new drug development and personalized cell therapies. Despite establishing of chemically defined hESC culture media over the past decade, these media's reliance on growth factors, which are costly and prone to degradation, poses a challenge for sustained and stable cell culture. Here, we introduce an hESC culture system(E6Bs) that facilitates the long-term, genetically stable expansion of hESCs, enabling cells to consistently sustain high levels of pluripotency markers, including NANOG, SOX2, TRA-1–60, and SSEA4, across extended periods. Moreover, organoids derived from hESCs using this medium were successfully established and expanded for at least one month, exhibiting differentiation into cortical organoids, GABAergic precursor organoids and heart-forming organoids. This innovative system offers a robust tool for preserving hESC homeostasis and modeling the nervous system in vitro.

**Supplementary Information:**

The online version contains supplementary material available at 10.1186/s13619-024-00220-y.

## Background

Human embryonic stem cells (hESCs) are distinguished by their remarkable ability for indefinite self-renewal and their potential to differentiate into diverse cell types (Thomson et al. 1998). The H9 cell line is particularly noteworthy, as it can differentiate into human neuroectoderm and brain organoids (Liu et al. 2015; Estridge et al. 2023), serving as an invaluable model for studying human neural development and enhancing drug discovery efforts (Lipsitz et al. 2018; Jinhong et al. 2021; Yang et al. 2015). Traditional culture conditions for hESCs faced significant challenges, including complex formulations and the risk of cross-species contamination (Sun et al. 2009), highlighting the necessity for chemically defined culture environments. Despite the introduction of a chemically defined medium (E8) over a decade ago, its key growth factors, such as TGF-β and bFGF, degrade rapidly and remain stable for only one week at 4 °C (Chen et al. 2011; Kuo et al. 2020). This rapid degradation compromises the quality of cell cultures. Recent advancements, including the AKIT medium, have attempted to improve culture conditions by eliminating growth factors; however, hESCs demonstrate reduced survival and growth rates in AKIT compared to E8 (Yasuda et al. 2018), indicating a need for further optimization of culture media to maintain hESC growth efficiency.

Saponins, renowned for their medicinal properties, contribute positively to tissue repair (Song and Xu 2009). They promote wound healing through activation of the TGF-β pathway, inhibit growth arrest and fibrosis via Smad and non-Smad pathways, and enhance angiogenesis by upregulating bFGF mRNA levels through the Wnt/β-catenin pathway (Kanzaki et al. 1998; Choi et al. 2014; Zhu et al. 2021). Furthermore, saponins have been found to support hESC proliferation, directed neural induction, and in vitro expansion, while also inhibiting neural cell apoptosis (Wang et al. 2007; Chu-Rong et al. 2008; Zhou et al. 2010; Si et al.^ 2011^; Zhang et al. 2018a; Tang et al. 2010; Wang et al. 2015). Among various types of saponins, those derived from quillaja bark (SQb) are chemically stable, have excellent adjuvant properties, and are extensively utilized in food additives and vaccines (Rajput et al. 2007; Reed et al. 2023; Barhate et al. 2013; Rönnberg et al. 1995; Fleck et al. 2019; Zhang et al. 2018b). Their aglycone components can modify cell membrane structures, providing protection against microbial infections, exhibiting antiviral activity both in vitro and in vivo, and reducing oxidative stress (Bangham and Horne 1962; Roner et al. 2007; Tam and Roner 2011; Abdel-Reheim et al. 2022; Ahmed Abdel-Reheim et al. 2017).

In this context, we propose an innovative hESC culture system that substitutes the key growth factors, bFGF and TGF-β in E8, with saponins from the soapbark tree. This approach facilitates stable hESC expansion (over 15 passages) and directs in vitro differentiation into cortical organoids, GABAergic precursor organoids and heart-forming organoids.

## Results

### SQb enhances survival on short-term hESC culture

**Fig. 1:**
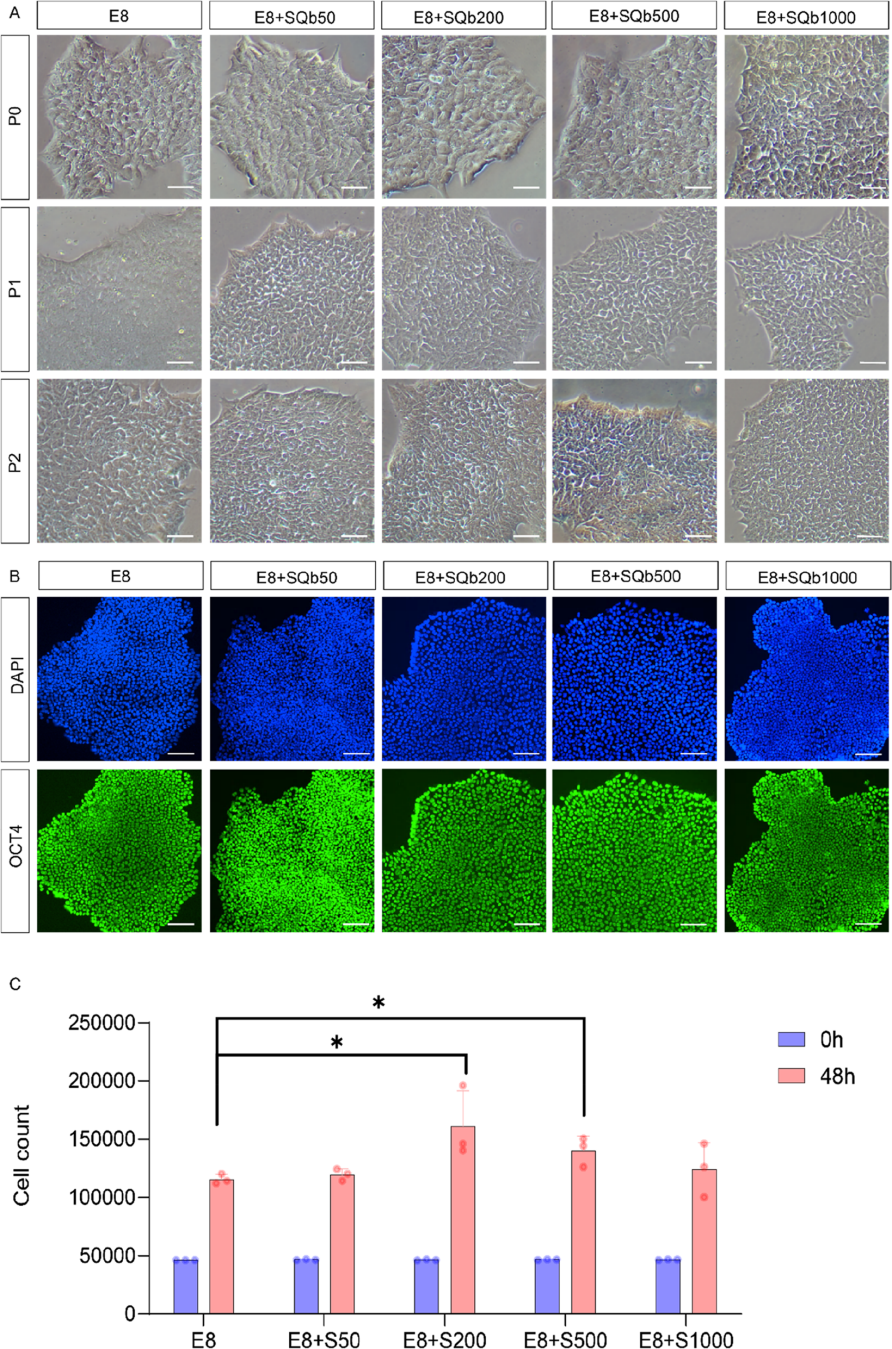
SQb promotes the proliferation of hESCs. **A** H9 cells were cultured in E8 medium with varying concentrations of SQb (50 ng/mL to 1000 ng/mL). Scale bar: 50 μm. **B** Immunostaining for pluripotent markers in H9 cells (passage 2) cultured with different concentrations of SQb in E8 medium. Scale bar: 50 μm. **C** The number of surviving H9 cells in E8 with various concentrations of SQb (50 ng/mL to 1000 ng/mL) after 48 h. ****P < 0.0001; *n* = 3. Values are the mean ± SD of three independent experiments

hESCs are highly sensitive to their growth conditions, necessitating a culture medium that is both safe and stable for medical applications. We initially assessed the toxicity of SQb on hESCs. Our results indicated that SQb concentrations ranging from 50 ng/mL to 1000 ng/mL did not induce cell death in hESCs (Fig. 1A). After two passages, the cell colonies maintained their typical undifferentiated morphology with distinct boundaries. Immunostaining for OCT4, a pluripotency marker, revealed high expression levels in these cells, as depicted in Fig. 1B. Furthermore, we evaluated the survival of H9 cells at various SQb concentrations in E8 medium after 48 h. Optimal survival effects were observed at 200 ng/mL and 500 ng/mL, indicating that SQb can enhance hESC survival (Fig. 1C).

### SQb can replace TGF-β in E8 culture system

**Fig. 2:**
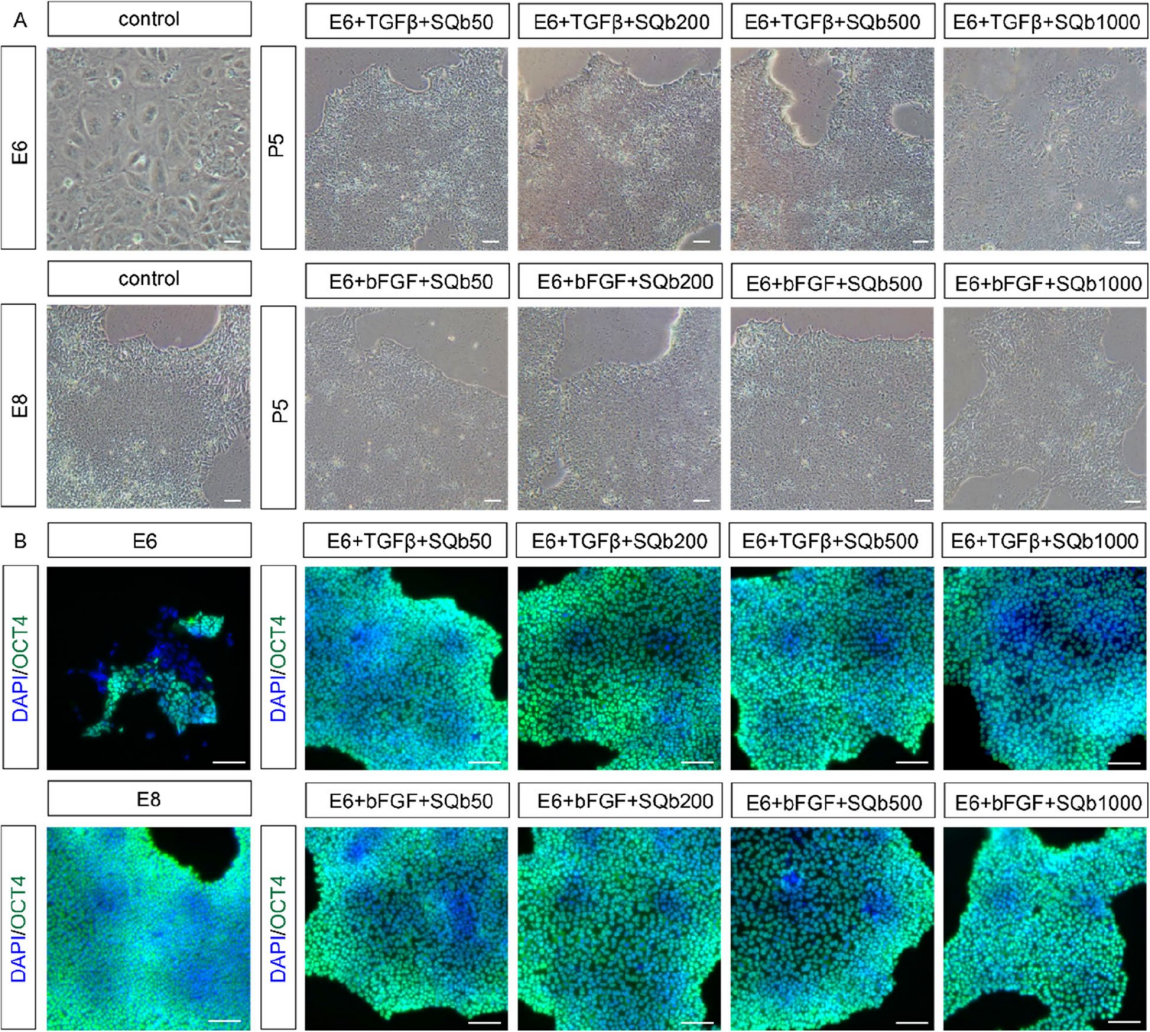
SQb can replace either bFGF or TGFβ in E8 system for short-term culture. **A** H9 cells were cultured for 5 passages in E8 medium with varying concentrations of SQb, either without bFGF or without TGFβ. Scale bar: 50 μm. **B** Immunostaining for pluripotent markers in H9 cells cultured for 5 passages in E8 medium with different concentrations of SQb, either without bFGF or without TGFβ. Scale bar: 50 μm

To improve the stability of the culture medium, we explored whether SQb could replace critical growth factors such as bFGF and TGF-β in E8 medium. We tested SQb concentrations of 50, 200, 500, and 1000 ng/mL to replace either bFGF or TGF-β, comparing these to the standard E8 control group (Fig. 2A). In short-term cultures (up to passage 5), SQb successfully supported hESC proliferation and maintained intact colonies without differentiation. Conversely, E8 medium lacking bFGF and TGF-β, or E6 medium supplemented with only 50 ng/mL bFGF, led to cell differentiation. Immunostaining showed that OCT4 was expressed in hESCs treated with SQb at 50, 200, 500, and 1000 ng/mL, replacing either bFGF or TGF-β (Fig. 2B). Based on these observations, 200 ng/mL was selected as the optimal SQb concentration to promote cell proliferation (Fig. 1A).

**Fig. 3:**
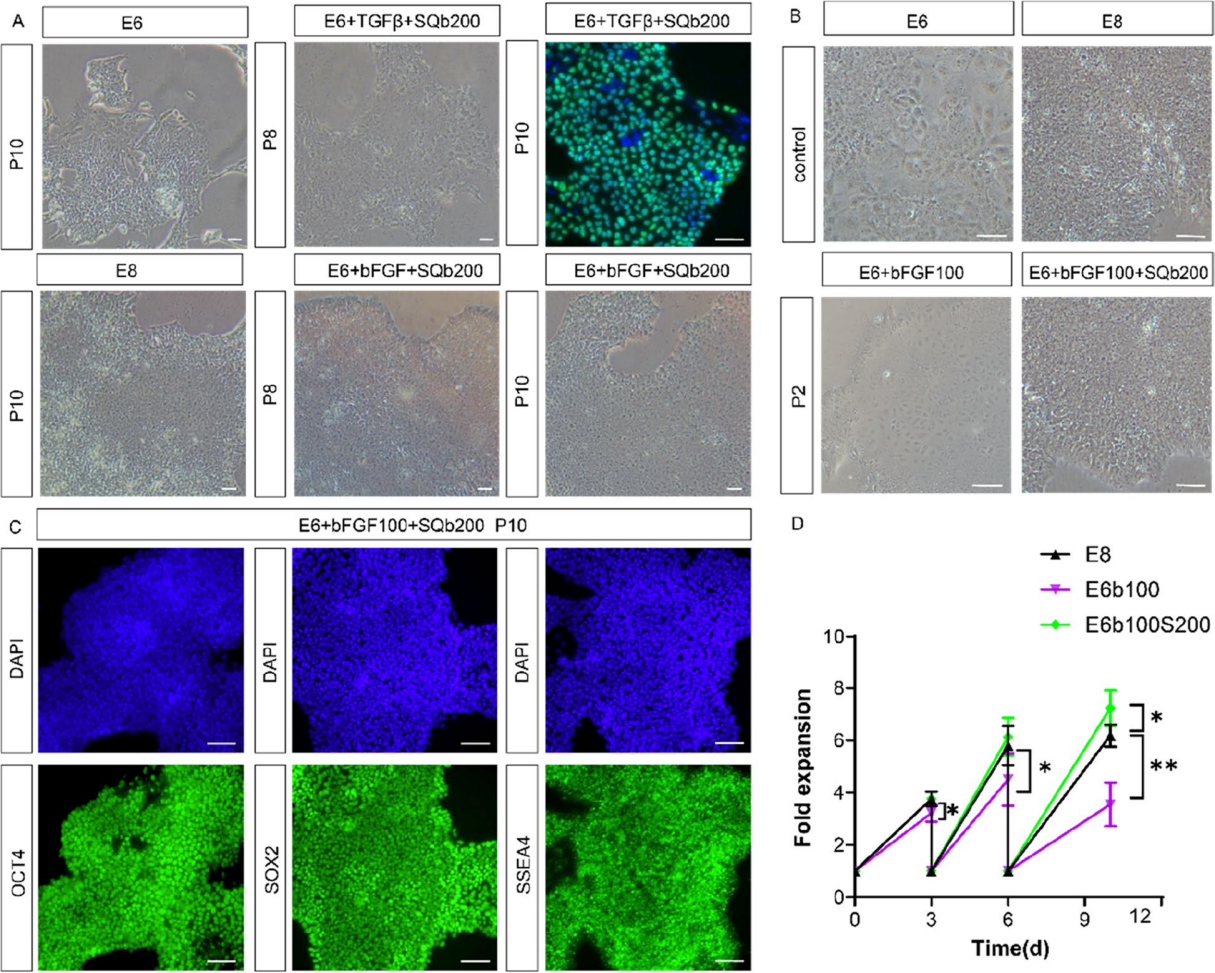
SQb can replace either bFGF or TGF-β in E8 system for long-term culture. **A** H9 cells were cultured for 10 passages in E8 medium where SQb replaced either bFGF or TGF-β. Scale bar: 50 μm. **B** Brightfield images of H9 cells cultured under different conditions. Scale bar: 50 μm. **C** Immunostaining for pluripotent markers in H9 cells cultured with SQb replacing bFGF or TGF-β in E6 medium for 10 passages. Scale bar: 50 μm. **D** Fold expansion of H9 cells in the indicated medium (200,000 cells were plated at each passage). *P < 0.05, **P < 0.01; *n* = 3. Values are the mean ± SD of three independent experiments

We further investigated the long-term effects of using 200 ng/mL SQb to replace either bFGF or TGF-β in the E6 culture system. After 10 passages, hESCs sustained expansion when TGF-β was replaced by SQb. However, in the absence of bFGF, some OCT4 expression was lost over time (Fig. 3A). Without TGF-β, hPSCs differentiated after two passages, but with the addition of 200 ng/mL SQb, hPSCs maintained proliferation without differentiation (Fig. 3B). Replacing TGF-β with SQb resulted in higher cell proliferation compared to the standard E8 medium (Fig. 3D). To confirm SQb's ability to maintain pluripotency in long-term culture, we examined the expression of pluripotent stem cell markers such as OCT4, SOX2, and SSEA-4 in cells cultured in E6 medium with bFGF and 200 ng/mL SQb (Fig. 3C, D).

### SQb reduces the required dosage of bFGF in E8 medium

**Fig. 4:**
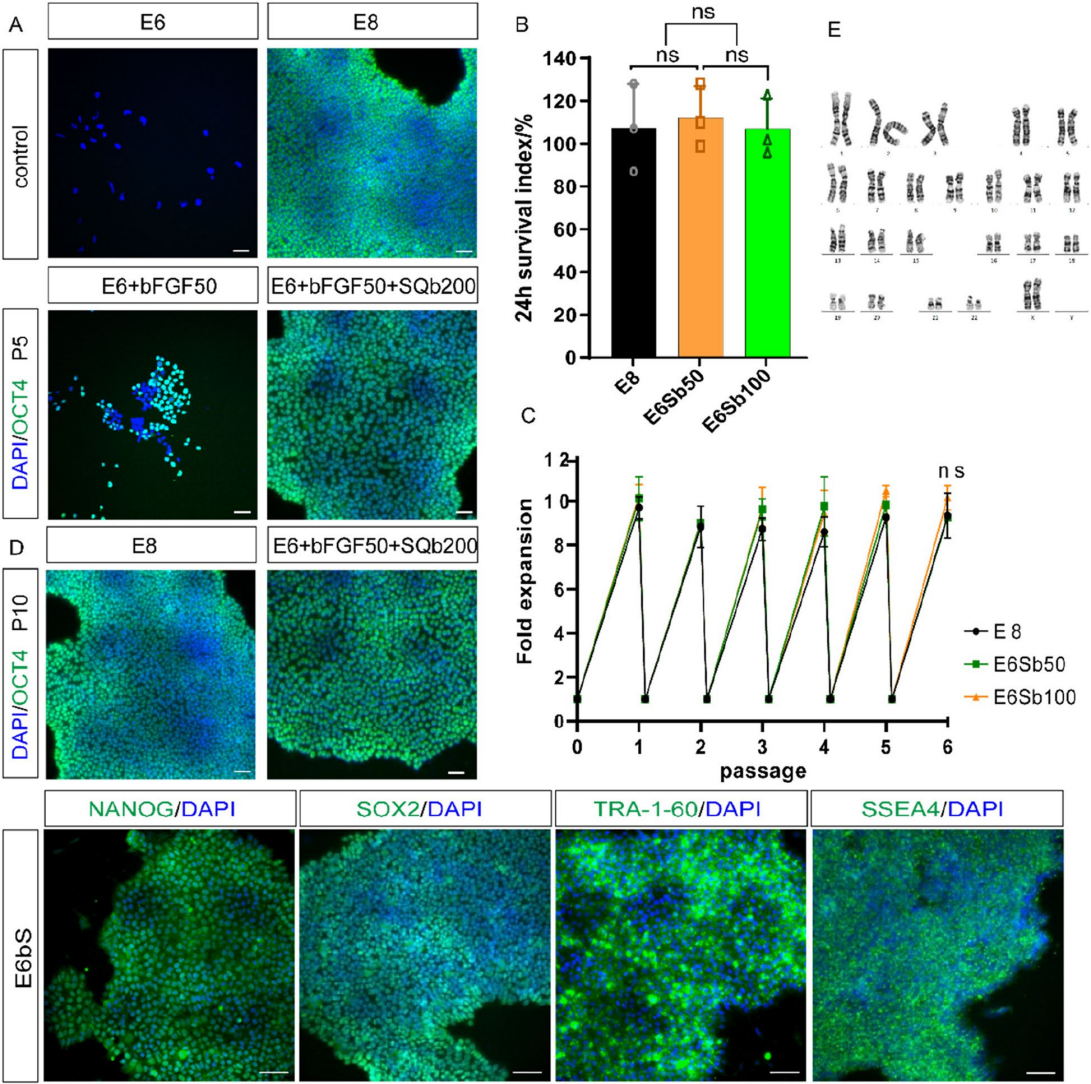
SQb replaces TGF-β and half the dosage of bFGF in E8 medium for long-term culture. **A** Immunostaining for pluripotent markers in H9 cells cultured under different conditions for 5 passages. Scale bar: 50 μm. **B** 24 h survival index of H9 cells in E6Sb50 medium compared to E8 and E6Sb100 medium. The 'survival index' represents the number of surviving cells divided by the number of input cells. *n* = 3, values are the mean ± SD of three independent experiments. **C** Fold expansion of hESCs in the indicated medium (200,000 cells were plated at each passage), *n* = 3. Values are the mean ± SD of three independent experiments. **D** Immunostaining for pluripotent markers in H9 cells cultured in E6bS medium for 10 passages. Scale bar: 50 μm. **E** Standard G-banding karyotyping results for H9 cells cultured in E6bE medium

Although SQb could not completely replace bFGF in the E8 culture system (Fig. 3A), it demonstrated the potential to reduce the required dosage of bFGF. To investigate this, we supplemented E6 medium with 200 ng/mL SQb and varied the bFGF concentration: 100 ng/mL in E6Sb100 and a reduced dose of 50 ng/mL in E6Sb50. A 24-h cell survival index assay revealed that 50 ng/mL bFGF achieved the same survival rate as 100 ng/mL (Fig. 4B). Further, we assessed the expansion rate of hPSCs in both E6Sb100 and E6Sb50 media and found comparable proliferation rates to that observed in E8 medium (Fig. 4C). OCT4 expression was consistently maintained in cells cultured in E6Sb50. In contrast, cells cultured with 50 ng/mL bFGF in E6 medium gradually lost OCT4 expression, leading to differentiation (Fig. 4A). Long-term culture in E6Sb50 (termed E6Sb) preserved the expression of pluripotency markers including OCT4, NANOG, SOX2, SSEA-4, and TRA-1–60 (Fig. 4C, D). To certificate that chromosomal mutations didn’t occur during hESCs culture, we performed karyotype analysis on the E6bS-treated H9 cells using the G-banding method (Wang et al. 2022a). The analysis revealed a normal chromosomal number of 46 chromosomes, with an XX sex chromosome composition, indicating a female karyotype. No significant chromosomal abnormalities were observed (Fig. 4E).

### Gene expression profiles in E6bS medium compared to E8

**Fig. 5:**
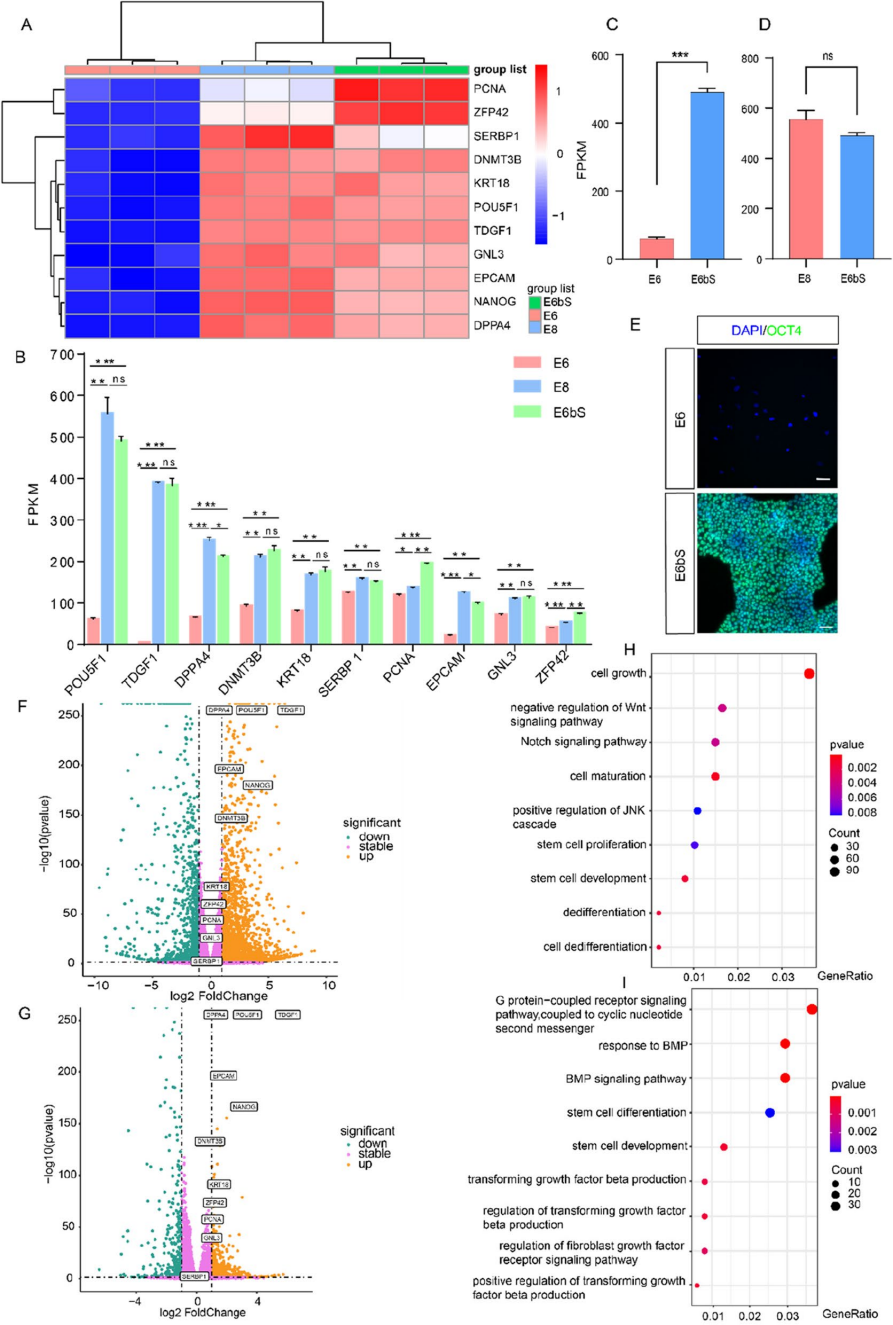
Gene expression in hESCs cultured in E6bS is similar to that in E8. **A** Heatmap of differential gene expression in E6bS-treated groups compared to E8 and E6 groups, *n* = 3, means ± SEM. **B** FPKM values of genes associated with stem cell pluripotency and proliferation, *n* = 3, means ± SEM. *P ≤ 0.05, ** *P* ≤ 0.01, *** *P* ≤ 0.001. **C** and **D** FPKM values of genes associated with stem cell pluripotency, *n* = 3, means ± SEM, *** *P* ≤ 0.001. **E** Immunostaining for pluripotent markers in H9 cells cultured in E6bS medium compared to those in E6 medium. Scale bar: 50 μm. **F** Volcano plot of differential gene expression in E6bS-treated groups compared to E6-treated groups. **G** Volcano plot of differential gene expression in E6bS-treated groups compared to E8-treated groups. **H** Bubble chart of cell proliferation-related pathways in E6bS-treated groups compared to E6-treated groups. **I** Bubble chart of cell proliferation-related pathways in E6bS-treated groups compared to E8-treated groups

RNA sequencing was performed on hESCs cultured in E6bS, E8, and E6 control groups to compare gene expression. EPCAM is associated with tissue plasticity regulation and stem cell differentiation (Fagotto 2020; Sasaki et al. 2015). DPPA4 is involved in embryonic and stem cell development and the activation of the POU5F1, SOX2, and NANOG pathways (Eckersley-Maslin et al. 2019; Gretarsson et al. 2020). SERBP1 participates in mRNA stability and modification (Lebensohn et al. 2016). PCNA, as a nuclear proliferation antigen, is involved in DNA unwinding and plays a role in stress responses and stem cell pluripotency (Wang et al. 2022b). ZFP42, as a pluripotency marker gene, participates in the DPPA4 and NANOG pathways (Dehghanian et al. 2024; Meek et al. 2020).The E6bS group exhibited 3,719 differentially expressed genes (DEGs) compared to the E6 control, with 66.5% up-regulated. Compared to the E8 group, 1,177 DEGs were observed in E6bS, with 71.8% up-regulated (Fig. 5A; Table S1). There were no significant differences in the expression of key pluripotency markers, such as POU5F1 (OCT4), TDGF1, DNMT3B, KRT18, SERBP1, and GNL3, between E6bS and E8 groups (Fig. 5B). Notably, POU5F1 expression in E6bS differed significantly from E6 but was similar to E8 (Fig. 5C-E). Volcano plot analysis highlighted that stem cell-specific genes like NANOG, POU5F1, and DPPA4 were significantly up-regulated in E6bS compared to E6, with no significant difference from E8 (Fig. 5F, G). Gene Ontology (GO) analysis revealed that genes associated with cell proliferation pathways were significantly up-regulated in E6bS compared to both E6 and E8 (Fig. 5H, I).

### Directed differentiation capacity of hPSCs cultured in E6bS

**Fig. 6:**
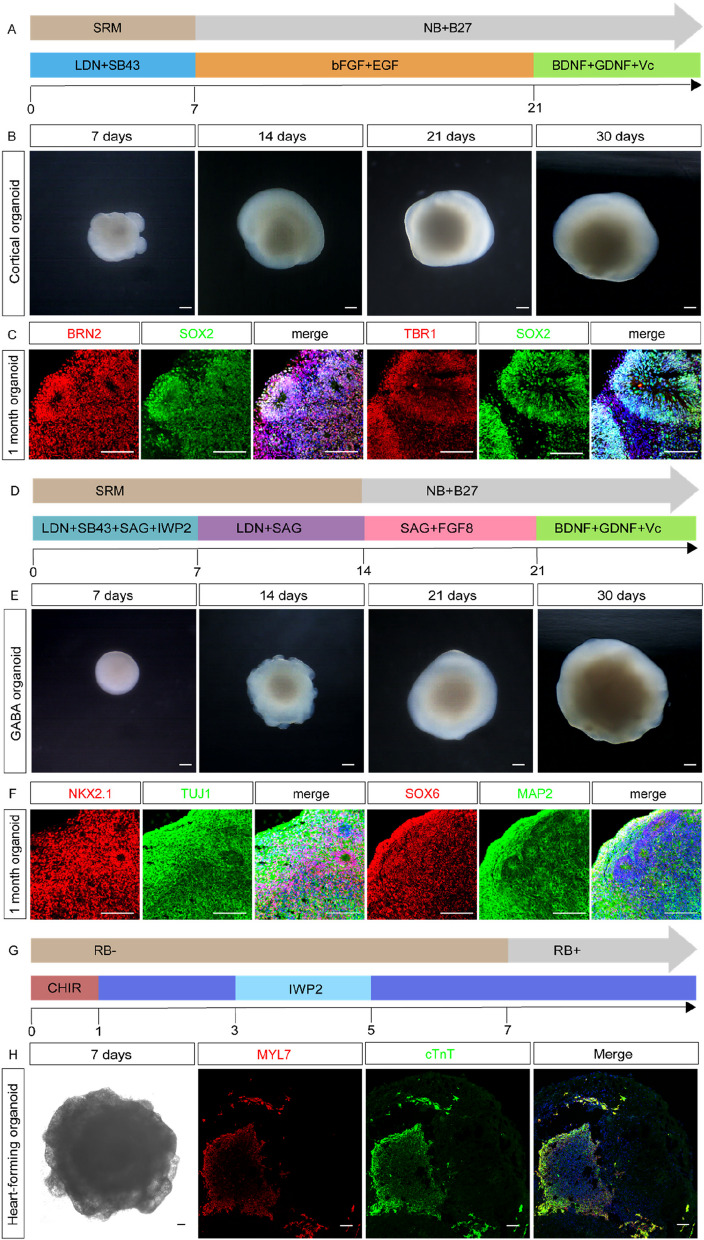
The E6bS culture system does not affect the directed differentiation ability of stem cells. **A** Schematic diagram of cortical organoids induce. **B** Differentiation of cortical organoids from H9 cells cultured in E6bS medium. Scale bar: 50 µm. **C** Four-week differentiation of cortical organoids from H9 cells cultured in E6bS medium, showing cortical layers marked by TBR1 + , BRN2 + , and SOX2 + neural progenitor cells. Scale bar: 50 µm. **D** Schematic diagram of GABA precursor organoid induction. **E** Differentiation of GABA precursor organoids from H9 cells cultured in E6bS medium. Scale bar: 50 µm. **F** Four-week differentiation of GABA organoids from H9 cells cultured in E6bS medium, showing NKX2.1 + /TUJ1 + and SOX6 + /MAP2 + inhibitory progenitor cells and inhibitory interneurons. Scale bar: 50 µm. **G** Schematic diagram of heart-forming organoid induction. **H** Differentiation of heart-forming organoids from H9 cells cultured in E6bS medium, showing MYL7 + /cTnT + cardiomyocytes. Scale bar: 100 µm

The directed differentiation capabilities of hPSCs cultured in E6bS were evaluated by inducing the formation of cortical and GABA precursor organoids. After one-month, cortical organoids were analyzed through immunofluorescence, displaying markers such as TBR1 and BRN2 and ventricular-zone-like (VZ-like) structures containing SOX2 + neural progenitor cells (Fig. 6A-C). Similarly, GABA precursor organoids, cultured for 30 days, were examined for markers of inhibitory progenitor cells, including NKX2.1 and SOX6, which co-expressed with MAP2, indicating the potential for differentiation into inhibitory interneurons (Fig. 6D-F). Additionally, through the induction of cardiomyocyte organoids, we also demonstrated the cells' potential for differentiation into the mesodermal lineage (Fig. 6G, H). Taken together, these findings demonstrate that the differentiation potential of hPSCs in the E6bS culture system remains intact.

## Discussion

E8 medium is a well-established culture system for hESCs under defined conditions, faces challenges due to the high cost and instability of key components such as bFGF and TGF-β (Chen et al. 2011; Kuo et al. 2020). Our research demonstrates that SQb can effectively replace TGF-β and reduce the required dosage of bFGF for hESC expansion. We have developed a novel culture system, E6bS, which supports the long-term culture of hPSCs while maintaining their pluripotency. This innovative system has the potential to significantly reduce the costs associated with the production of hESCs and their derivatives for medical applications and basic research.

Directed differentiation into organoids would effectively showcase the differentiation potential of cells under physiological conditions. Therefore, we employed our laboratory's well-established method for inducing cortical organoids and GABAergic precursor organoids for validation. To further confirm that hESCs cultured in the E6bS system can be directed to differentiate into other germ layer cells, we also used a cardiac organoid induction method for H9 cells after E6bS culture. Following the induction protocol by Drakhlis et al. (2021), we observed beating cardiomyocytes on day 7 and identified myocardial tissue through immunofluorescence staining (Fig.6H; Video S1). These results demonstrate that human embryonic stem cells cultured in the E6bS system retain the potential to differentiate into mesodermal lineage cells. The differentiation potential of endodermal cells can be further validated in the future through endodermal organoid induction methods.

Previous studies have indicated that the TGFβ/Smad and FGF/MAP signaling pathways collaborate to suppress BMP signaling, sustain the expression of pluripotency markers such as NANOG, OCT4, and SOX2, and promote the long-term undifferentiated proliferation of human ESCs (Xu et al. 2008). TGFβ/Smad signaling is essential for the self-renewal of ESCs, primarily by directly interacting with the NANOG promoter. Our findings suggest that replacing TGFβ entirely with SQb also maintains NANOG expression (Fig.5A). Furthermore, when cells were cultured with SQb and half the dose of bFGF, we observed an enhanced BMP signaling activity compared to the TGFβ/bFGF combination (Fig. 5I). These results imply that SQb may interact with the BMP pathway in a manner similar to TGFβ, influencing the expression of NANOG. In our study, the expression levels of EPCAM, DPPA4, and SERBP1 were slightly lower in E6bS medium compared to E8 medium, while the expression levels of PCNA and ZFP42 were higher in E6bS medium than in E8 medium (Fig. 5A). These findings suggest that E6bS medium may primarily affect specific pathways during stem cell culture. However, due to the complexity of the SQb component, further in-depth component analysis is needed to fully elucidate its impact on the BMP and NANOG signaling pathways.

The cost of E6bS medium is significantly lower than that of E8, primarily due to the reduced need for bFGF and TGF-β. While E8 medium contains TGF-β at 1.74 µg/L and bFGF at 100 µg/L, E6bS uses SQb at 200 µg/L and a reduced bFGF amount of 50 µg/L, resulting in a cost reduction for these growth factors. Additionally, the E6bS medium proves more practical for hPSC culture. We observed that SQb is stable at 4 °C for up to two months, unlike TGF-β and bFGF, which degrade within a week. When stored at -20 °C, SQb remains effective for at least two months. This enhanced stability minimizes waste associated with the degradation of growth factors in large-scale stem cell cultures.

In summary, our results indicate that SQb not only enhances hPSC proliferation and replaces TGF-β but also lowers the dosage of bFGF required for expansion. The E6bS system supports long-term hESC culture while maintaining pluripotency, and the gene expression profiles of hESCs cultured in E6bS are comparable to those in the E8 system. These advancements will benefit both basic research and clinical applications of hPSCs in the future.

## Methods

### Cell culture

Human embryonic stem cells (H9, purchased from WiCell, 22-W0510) were cultured on Matrigel-coated plates (Corning, 365230) using E8 medium, including 100 ng/ml bFGF (Peprotech), 1.74 ng/ml TGFβ and E6 medium which contains DMEM/F12 (Catalog # 11330032 Gibco) supplement with 13.6 µg/ml sodium selenium (Sigma), 1 mg/ml sodium chloride (Sigma), 64 µg/ml L Ascorbic acid 2-phosphate (Sigma), 20 µg/ml recombinant human insulin (Sigma) and 10 µg/ml human holo-transferrin (Sigma), 1% penicillin–streptomycin (Thermo Fisher Scientific). The modified medium, referred to as E6, excludes 100 ng/mL bFGF (Peprotech, AF10018B) and 1.74 ng/mL TGFβ (Proteintech, HZ1011). The E6 medium supplemented with 50 ng/mL bFGF, 1.74 ng/mL TGFβ, and 200 ng/mL saponins from quillaja bark (SQb, Sigma Aldrich, 8047152) is termed the E6bS system. Cultures were maintained at 37 °C in a humidified atmosphere with 5% CO_2_. For the subculture, hESCs were rinsed twice with DPBS (Gibco), and detached as single cells with TrypLE (Catalog # 12,563, Invitrogen) at 37 °C for 1–2 min and replated on Matrigel-coated plates. Change the fresh medium daily and cells are passaged every four to six days.

### Generation of cortical organoids from H9 cultured in the E6bS system

For cortical organoid formation, H9 cells were seeded at a density of 5 × 10^4^ cells per well in an ultra-low attachment 96-well plate on day 0 and cultured in SRM medium. This medium was supplemented with LDN193189 (100 nM, Sigma, SML0559) and SB431542 (10 nM, MCE, HY10431) from day 0 to day 7. To enhance cell survival during the initial 2 to 3 days post-seeding, ROCK inhibitor Y27632 (10 nM, Selleckchem, S1049) was added. On day 7, the culture medium was switched to Neurobasal medium (Gibco, 21103049) enriched with 1% penicillin–streptomycin (Gibco, 15140122), 1% Non-Essential Amino Acids (Gibco, 11140500), 1% GlutaMax (Gibco, 35050061), 2% B27 (Gibco, 17504044), 20 ng/mL bFGF, and 20 ng/mL EGF (Peprotech, 10047). Starting from day 21, spheroids were embedded in Matrigel, with medium changes to Neurobasal medium supplemented with 1% penicillin–streptomycin, 1% Non-Essential Amino Acids, 1% GlutaMax, 2% B27, 200 nM l-ascorbic acid 2-phosphate, 10 ng/mL BDNF (Peprotech, AF45002), and 10 ng/mL GDNF (Peprotech, AF45010). The spheroids were transferred to an ultra-low attachment 60 mm dish on day 25.

### Generation of GABA organoids from H9 cultured in the E6bS system

For GABA organoid generation, H9 cells were similarly seeded at 5 × 10^4^ cells per well in an ultra-low attachment 96-well plate on day 0 and cultured with SRM medium containing LDN193189 (100 nM), SB431542 (10 nM), IWP2 (10 nM, MCE, HY13912), and SAG (100 nM, MCE, HY12848) from day 0 to day 7. ROCK inhibitor Y27632 was again used to aid cell survival during the initial days post-seeding. On day 7, the medium was switched to SRM containing SAG (100 nM) and LDN193189 (100 nM). By day 14, the culture medium was changed to Neurobasal medium with 1% penicillin–streptomycin, 1% Non-Essential Amino Acids, 1% GlutaMax, SAG (100 nM), and FGF8 (100 nM, MCE, HYP7346). From day 21, the spheroids were embedded in Matrigel, and the medium was subsequently changed to Neurobasal medium supplemented with 1% penicillin–streptomycin, 1% Non-Essential Amino Acids, 1% GlutaMax, 2% B27, 200 nM l-ascorbic acid 2-phosphate, 10 ng/mL BDNF, and 10 ng/mL GDNF. Spheroids were moved to an ultra-low attachment 60 mm dish on day 25.

### Generation of Heart-forming organoids from H9 cultured in the E6bS system

For Heart-forming organoid generation, H9 cells were similarly seeded at 5,000 cells per well in an ultra-low attachment 96-well plate on day − 4 and cultured in E6bS (E8) medium containing 10 µM Y-27632. The plate was centrifuged at 300 g and 4 °C for 3 min, and the cells were incubated overnight to allow the formation of aggregates. On day − 2, the aggregates were embedded in a Matrigel droplet and incubated for 1 h to solidify the Matrigel. Following this, E6bS medium was added to the aggregates. Differentiation of these aggregates was initiated on day 0 by replacing the medium with RPMI/1640 medium containing 2% B-27 supplement without insulin (RB −), and supplemented with 7.5 µM CHIR. After 24 h, the medium was exchanged by RB − . On day 3, RB − supplemented with 5 µM IWP2 was added for 48 h, and the medium was exchanged again on day 5. From day 7 onwards, the aggregates were cultivated in RPMI/1640 medium containing 2% B-27 supplement (RB +). Differentiation was completed by day 10. HFOs were analyzed on day 3, with pictures of the whole HFOs taken using an Axio Observer A1 (Zeiss) or an Olympus CKX41 inverted microscope (Olympus).

### Immunostaining

Human embryonic stem cells (H9) cultured in plates were washed twice with PBS before fixation with 4% paraformaldehyde (PFA) for 20 min at room temperature, followed by three PBS washes. Blocking was conducted using 3% BSA with 0.3% Triton X-100 for 30 min at room temperature. Primary antibodies—mouse anti-OCT4 (1:2000, BD Pharmingen, 561465), mouse anti-SOX2 (1:50, BD Pharmingen, 561469), mouse anti-NANOG (1:100, BD Pharmingen, 560109), mouse anti-SSEA4 (1:100, Millipore, MAB4304), and mouse anti-TRA-1–60 (1:100, Millipore, MAB4360)—were incubated in 3% BSA with 0.3% Triton X-100 overnight at 4 °C. After three washes with PBS, secondary antibodies (anti-mouse Alexa 488, Life Technologies) and 4′,6-diamidino-2-phenylindole (DAPI) (Sigma, MBD0015) were applied and incubated for 1 h at room temperature, followed by three washes in 1 × PBS. Images were captured using a Nikon Confocal AIR-MP fluorescence confocal microscope.

Cortical and GABA organoids were fixed overnight at 4 °C in 4% PFA and subsequently immersed in 30% sucrose. Immunofluorescence involved 25 µm to 30 µm sections prepared using a cryostat. Sections were blocked in 3% BSA with 0.3% Triton X-100 for 60 min at room temperature. Primary antibodies—rabbit anti-NKX2.1 (1:300, Millipore, MAB5460), mouse anti-TUJ1 (1:1000, Covance, MS-435P), rabbit anti-SOX6 (1:1000, Sigma, ab5805), mouse anti-SOX2 (1:50, BD Pharmingen, 561469), rabbit anti-TBR1 (1:500, Abcam, ab31940), and rabbit anti-BRN2 (1:500, GeneTex, GTX114650)—were incubated overnight at 4 °C. Slides were washed three times for 10 min each in 1 × PBS on a shaker to remove excess primary antibody. Secondary antibodies—anti-mouse Alexa 488 (Invitrogen, A11001) and anti-rabbit Alexa 594 (Invitrogen, A11012)—along with DAPI were added at a 1:500 dilution and incubated at room temperature for 1 h. Following three PBS washes, images were captured using a Nikon Confocal AIR-MP fluorescence confocal microscope.

Heart-forming organoids were stained using the same method, with primary antibodies being cTnT (1:500, Thermo, MA512960) and MYL7 (1:500, proteintech, 17283–1-AP). Secondary antibodies included anti-mouse Alexa 488 (Invitrogen, A11001), anti-rabbit Alexa 594 (Invitrogen, A11012), and DAPI.

### RNA-sequencing

To explore the mechanism by which SQb enhances stem cell pluripotency and proliferation, we conducted RNA-sequencing with three biological replicates across three treatment groups: E6bS, E8, and E6. RNA purity, quantity, and integrity were assessed using the Agilent 2100 Bioanalyzer (Agilent Technologies, Santa Clara, CA, USA). cDNA libraries were prepared following the TruSeq Stranded mRNA LT Sample Prep Kit instructions (Illumina, San Diego, CA, USA) and sequenced on an Illumina NovaSeq 6000 platform to produce 150 bp paired-end reads. Initial data processing was performed using Trimmomatic to obtain approximately 50 million clean reads, which were then mapped to the human genome with HISAT2. Gene expression quantification was achieved using the Cufflinks method to calculate FPKM values, with read counts obtained through HTSeq-count. PCA analysis was executed using R (v 3.2.0) to verify sample reproducibility. Differential expression analysis was conducted using DESeq2, setting significance thresholds at a Q value < 0.05 and a fold change > 2 or < 0.5. DEGs were subjected to hierarchical clustering and GO enrichment analysis to delineate gene expression profiles.

### Karyotyping analysis

Colcemid (Thermo Fisher Scientific, 15212012, 50 ng/mL) was applied to H9 cultured in E6bS for two to six hours. Digested single cells were centrifuged, collected, and given one PBS wash. Pellets were resuspended by 200 μL DMEM, and then were hypotonic treated gently with 10 mL hypotonic solution (0.075 M KCl) at 37 °C for 30 min, then cells were fixed with 1 ml fix solution (methanol: glacial acetic acid = 3: 1) at room temperature for another 5 min. After centrifuging, the pellets were resuspended in 6 mL of cold, fresh fix solution for 20 min. This step was repeated once. The pellets were then collected by centrifugation and resuspended by 200 μL cold fix solution. Chromosomes were spread by gravity as cell solution was dropped (10 μL/drop) onto glass slides that had been pre-cooled at -80 °C. Hoechst33342 was applied for karyotype observation under LSM780 Meta confocal microscope (Zeiss). The software KTa-003 was used for standard G-banding karyotype analyses (GeneDiagnostics, Inc).

### Statistics

Data are presented as the mean ± SEM from a minimum of three independent experiments. Significance levels are denoted as *P < 0.05, **P < 0.01, and ***P < 0.001, with the number of experiments (n) specified in the figure legends. Statistical evaluations were performed using a one-tailed, unpaired t-test.

## Supplementary Information

Supplementary Material 1: Table S1. Differentially Expressed Genes (DEGs) Identified in E6bS Medium Compared to E6 and E8 Controls.

Supplementary Material 2: Video S1. Visualization of Beating Cardiomyocytes Derived from H9 cells cultured in E6bS medium.

## Data Availability

The datasets generated during the current study are available in the National Genomics Data Center, ACCESSION NUMBER: HRA004262, https://ngdc.cncb.ac.cn/gsa-human/s/RbQKA2tI.

## Abbreviations

hESC: Human embryonic stem cell
SQb: Quillaja bark
H9: Human embryonic stem cells
PFA: Paraformaldehyde
VZ-like: Ventricular-zone-like
DEGs: Differentially expressed genes
GO: Gene Ontology

## References

[CR1] Abdel-Reheim MA, Ashour AA, Khattab MA, Gaafar AGA. Quillaja saponaria bark saponin attenuates methotrexate induced hepatic oxidative stress, inflammation and associated liver injury in rats. J Appl Pharm Sci. 2022;12(5):129–41. 10.7324/JAPS.2022.120510.

[CR2] Ahmed Abdel-Reheim M, Messiha BAS, Abo-Saif AA. Quillaja saponaria bark saponin protects Wistar rats against ferrous sulphate-induced oxidative and inflammatory liver damage. Pharm Biol. 2017;55(1):1972–83. 10.1080/13880209.2017.1345950. 28728456 10.1080/13880209.2017.1345950 PMC6130630

[CR3] Bangham A, Horne R. Action of saponin on biological cell membranes. Nature. 1962;196(4858):952–3. 10.1038/196952a0. 13966357 10.1038/196952a0

[CR4] Barhate G, Gautam M, Gairola S, Jadhav S, Pokharkar V. Quillaja saponaria extract as mucosal adjuvant with chitosan functionalized gold nanoparticles for mucosal vaccine delivery: stability and immunoefficiency studies. Int J Pharm. 2013;441(1–2):636–42. 10.1016/j.ijpharm.2012.10.033. 23117021 10.1016/j.ijpharm.2012.10.033

[CR5] Chen G, Gulbranson DR, Hou Z, Bolin JM, Ruotti V, Probasco MD, et al. Chemically defined conditions for human iPSC derivation and culture. Nat Methods. 2011;8(5):424–9. 10.1038/nmeth.1593. 21478862 10.1038/nmeth.1593 PMC3084903

[CR6] Choi JH, Hwang YP, Kim HG, Khanal T, Do MT, Jin SW, et al. Saponins from the roots of Platycodon grandiflorum suppresses TGFβ1-induced epithelial-mesenchymal transition via repression of PI3K/Akt, ERK1/2 and Smad2/3 pathway in human lung carcinoma A549 cells. Nutr Cancer. 2014;66(1):140–51. 10.1080/01635581.2014.853087. 24341702 10.1080/01635581.2014.853087

[CR7] Chu-Rong W, Yi S, Jian-Ping Z (2008) Effects of panax notoginseng saponing on activity and differentiation of neonate rat hippocampus neural stem cells in vitro. J Jinggangshan Univ

[CR8] Dehghanian F, Bovio PP, Gather F, Probst S, Naghsh-Nilchi A, Vogel T. ZFP982 confers mouse embryonic stem cell characteristics by regulating expression of Nanog, Zfp42, and Dppa3. Biochim Biophys Acta Mol Cell Res. 2024;1871(4):119686. 10.1016/j.bbamcr.2024.119686. 38342310 10.1016/j.bbamcr.2024.119686

[CR9] Drakhlis L, Biswanath S, Farr C-M, Lupanow V, Teske J, Ritzenhoff K, et al. Human heart-forming organoids recapitulate early heart and foregut development. Nat Biotechnol. 2021;39(6):737–46. 10.1038/s41587-021-00815-9. 33558697 10.1038/s41587-021-00815-9 PMC8192303

[CR10] Eckersley-Maslin M, Alda-Catalinas C, Blotenburg M, Kreibich E, Krueger C, Reik W. Dppa2 and Dppa4 directly regulate the Dux-driven zygotic transcriptional program. Genes Dev. 2019;33(3–4):194–208. 10.1101/gad.321174.118. 30692203 10.1101/gad.321174.118 PMC6362816

[CR11] Estridge RC, O’Neill JE, Keung AJ. Matrigel tunes H9 stem cell-derived human cerebral organoid development. Organoids. 2023;2(4):165–76. 10.3390/organoids2040013. 38196836 10.3390/organoids2040013 PMC10776236

[CR12] Fagotto F. EpCAM as modulator of tissue plasticity. Cells. 2020;9(9):2128. 10.3390/cells9092128. 32961790 10.3390/cells9092128 PMC7563481

[CR13] Fleck JD, Betti AH, Da Silva FP, Troian EA, Olivaro C, Ferreira F, et al. Saponins from Quillaja saponaria and Quillaja brasiliensis: particular chemical characteristics and biological activities. Molecules. 2019;24(1):171. 10.3390/molecules24010171. 30621160 10.3390/molecules24010171 PMC6337100

[CR14] Gretarsson KH, Hackett JA. Dppa2 and Dppa4 counteract de novo methylation to establish a permissive epigenome for development. Nat Struct Mol Biol. 2020;27(8):706–16. 10.1038/s41594-020-0445-1. 32572256 10.1038/s41594-020-0445-1

[CR15] Jinhong X, Shi F, Naweng W, Bo L, Yongheng H, Qi F, et al (2021) An efficient chemical-defined condition for generation of human induced pluripotent stem cells

[CR16] Kanzaki T, Morisaki N, Shiina R, Saito Y. Role of transforming growth factor-β pathway in the mechanism of wound healing by saponin from Ginseng Radix rubra. Br J Pharmacol. 1998;125(2):255–62. 10.1038/sj.bjp.0702052. 9786496 10.1038/sj.bjp.0702052 PMC1565613

[CR17] Kuo H-H, Gao X, DeKeyser J-M, Fetterman KA, Pinheiro EA, Weddle CJ, et al. Negligible-cost and weekend-free chemically defined human iPSC culture. Stem Cell Reports. 2020;14(2):256–70. 10.1016/j.stemcr.2019.12.007. 31928950 10.1016/j.stemcr.2019.12.007 PMC7013200

[CR18] Lebensohn AM, Dubey R, Neitzel LR, Tacchelly-Benites O, Yang E, Marceau CD, et al. Comparative genetic screens in human cells reveal new regulatory mechanisms in WNT signaling. Elife. 2016;5:e21459. 27996937 10.7554/eLife.21459 PMC5257257

[CR19] Lipsitz YY, Woodford C, Yin T, Hanna JH, Zandstra PW. Modulating cell state to enhance suspension expansion of human pluripotent stem cells. Proc Natl Acad Sci. 2018;115(25):6369–74. 10.1073/pnas.1714099115. 29866848 10.1073/pnas.1714099115 PMC6016797

[CR20] Liu A, Zhang D, Liu L, Gong J, Liu C. A simple method for differentiation of H9 cells into neuroectoderm. Tissue Cell. 2015;47(5):471–7. 10.1016/j.tice.2015.07.006. 26253416 10.1016/j.tice.2015.07.006

[CR21] Meek S, Wei J, Oh T, Watson T, Olavarrieta J, Sutherland L, et al. A stem cell reporter for investigating pluripotency and self-renewal in the rat. Stem Cell Reports. 2020;14(1):154–66. 10.1016/j.stemcr.2019.12.001. 31902707 10.1016/j.stemcr.2019.12.001 PMC6962659

[CR22] Rajput ZI, Hu SH, Xiao CW, Arijo AG. Adjuvant effects of saponins on animal immune responses. J Zhejiang Univ SciB. 2007;8(3):153–61. 10.1631/jzus.2007.B0153. 10.1631/jzus.2007.B0153 PMC1810383 17323426

[CR23] Reed J, Orme A, El-Demerdash A, Owen C, Martin LB, Misra RC, et al. Elucidation of the pathway for biosynthesis of saponin adjuvants from the soapbark tree. Science. 2023;379(6638):1252–64. 10.1126/science.adf3727. 36952412 10.1126/science.adf3727

[CR24] Roner MR, Sprayberry J, Spinks M, Dhanji S. Antiviral activity obtained from aqueous extracts of the Chilean soapbark tree (Quillaja saponaria Molina). J Gen Virol. 2007;88(1):275–85. 10.1099/vir.0.82321-0. 17170461 10.1099/vir.0.82321-0

[CR25] Rönnberg B, Fekadu M, Morein B. Adjuvant activity of non-toxic Quillaja saponaria Molina components for use in ISCOM matrix. Vaccine. 1995;13(14):1375–82. 10.1016/0264-410X(95)00105-A. 8585296 10.1016/0264-410x(95)00105-a

[CR26] Sasaki K, Yokobayashi S, Nakamura T, Okamoto I, Yabuta Y, Kurimoto K, et al. Robust in vitro induction of human germ cell fate from pluripotent stem cells. Cell Stem Cell. 2015;17(2):178–94. 10.1016/j.stem.2015.06.014. 26189426 10.1016/j.stem.2015.06.014

[CR27] Si Y-C, Zhang J-P, Xie C-E, Zhang L-J, Jiang X-N. Effects of Panax notoginseng saponins on proliferation and differentiation of rat hippocampal neural stem cells. Am J Chin Med. 2011;39(05):999–1013. 10.1142/S0192415X11009366. 21905288 10.1142/S0192415X11009366

[CR28] Song X, Hu S. Adjuvant activities of saponins from traditional Chinese medicinal herbs. Vaccine. 2009;27(36):4883–90. 10.1016/j.vaccine.2009.06.033. 19559122 10.1016/j.vaccine.2009.06.033

[CR29] Sun N, Panetta NJ, Gupta DM, Wilson KD, Lee A, Jia F, et al. Feeder-free derivation of induced pluripotent stem cells from adult human adipose stem cells. Proc Natl Acad Sci. 2009;106(37):15720–5. 10.1073/pnas.0908450106. 19805220 10.1073/pnas.0908450106 PMC2739869

[CR30] Tam KI, Roner MR. Characterization of in vivo anti-rotavirus activities of saponin extracts from Quillaja saponaria Molina. Antiviral Res. 2011;90(3):231–41. 10.1016/j.antiviral.2011.04.004. 21549151 10.1016/j.antiviral.2011.04.004 PMC3106224

[CR31] Tang Y, Huang X, Tan H, Chen B, Deng C. Effect of panax notoginseng saponins on neuronal apoptosis and mitochondrial apoptosis pathway expression c-Jun N-terminal kinase after cerebral ischemia-reperfusion in mice. Zhongguo Shiyan Fangjixue Zazhi. 2010;16(16):129-32,36.

[CR32] Thomson JA, Itskovitz-Eldor J, Shapiro SS, Waknitz MA, Swiergiel JJ, Marshall VS, et al. Embryonic stem cell lines derived from human blastocysts. Science. 1998;282(5391):1145–7. 10.1126/science.282.5391.1145. 9804556 10.1126/science.282.5391.1145

[CR33] Wang S, Li Y, Wang Y, Feng M. Effect of TSPG on proliferation and differentiation of human embryonic neural stem cell into dopaminergic neuron. Zhongguo Zhong Yao Za Zhi. 2007;32(13):1310–3. 17879733

[CR34] Wang B, Li Y, Li XP, Li Y. Panax notoginseng saponins improve recovery after spinal cord transection by upregulating neurotrophic factors. Neural Regen Res. 2015;10(8):1317–20. 10.4103/1673-5374.162766. 26487862 10.4103/1673-5374.162766 PMC4590247

[CR35] Wang L-B, Li Z-K, Wang L-Y, Xu K, Ji T-T, Mao Y-H, et al. A sustainable mouse karyotype created by programmed chromosome fusion. Science. 2022a;377(6609):967–75. 10.1126/science.abm1964. 36007034 10.1126/science.abm1964

[CR36] Wang W, Yan T, Guo X, Cai H, Liang C, Huang L, et al. KAP1 phosphorylation promotes the survival of neural stem cells after ischemia/reperfusion by maintaining the stability of PCNA. Stem Cell Res Ther. 2022b;13(1):290. 10.1186/s13287-022-02962-5. 35799276 10.1186/s13287-022-02962-5 PMC9264526

[CR37] Xu R-H, Sampsell-Barron TL, Gu F, Root S, Peck RM, Pan G, et al. NANOG is a direct target of TGFβ/activin-mediated SMAD signaling in human ESCs. Cell Stem Cell. 2008;3(2):196–206. 10.1016/j.stem.2008.07.001. 18682241 10.1016/j.stem.2008.07.001 PMC2758041

[CR38] Yang Y, Adachi K, Sheridan MA, Alexenko AP, Schust DJ, Schulz LC, et al. Heightened potency of human pluripotent stem cell lines created by transient BMP4 exposure. Proc Natl Acad Sci. 2015;112(18):E2337–46. 10.1073/pnas.1504778112. 25870291 10.1073/pnas.1504778112 PMC4426460

[CR39] Yasuda SY, Ikeda T, Shahsavarani H, Yoshida N, Nayer B, Hino M, et al. Chemically defined and growth-factor-free culture system for the expansion and derivation of human pluripotent stem cells. Nat Biomed Eng. 2018;2(3):173–82. 10.1038/s41551-018-0200-7. 31015717 10.1038/s41551-018-0200-7

[CR40] Zhang F-l, Yang L-j, Hu W-y, Zhou J-y, Ma S-x, Xiao Z-c. Effect of ginsenoside Rg3 on mouse neural stem cell differentiation in vitro. Chin J Tissue Eng Res. 2018a;22(13):2098.

[CR41] Zhang X-P, Li Y-D, Luo L-L, Liu Y-Q, Li Y, Guo C, et al. Astragalus saponins and liposome constitute an efficacious adjuvant formulation for cancer vaccines. Cancer Biother Radiopharm. 2018b;33(1):25–31. 10.1089/cbr.2017.2369. 29466034 10.1089/cbr.2017.2369

[CR42] Zhou Z, Wang X, Zhong P. Effects of Ginsenoside Rg_1 on the proliferation of neural stem cells cultured in vitro. Chin J Inform Tradit Chin Med. 2010;17:28-30.

[CR43] Zhu P, Jiang W, He S, Zhang T, Liao F, Liu D, et al. Panax notoginseng saponins promote endothelial progenitor cell angiogenesis via the Wnt/β-catenin pathway. BMC Complement Med Ther. 2021;21:1–11. 10.1186/s12906-021-03219-z. 33557814 10.1186/s12906-021-03219-z PMC7869233

